# Rules for the Proper Usage of Heraldry in the United States

**Published:** 1895-11

**Authors:** 


Rules for the Proper Usage of Heraldry in the United States, and other extracts from the popular authority, *Heraldry in America*. By Eugene Zieber. Published by The Department of Heraldry of The Bailey, Banks & Biddle Co., Philadelphia.

This beautiful monograph is published by the justly celebrated jewelry firm of Bailey, Banks & Biddle, of Philadelphia.

The motive for its publication may be found by perusal of the following extracts from the preface:

This volume is designed to meet a felt want in America for a popular work on heraldry. The writer has endeavored to group in a concise and intelligent manner all that is necessary to enable the student correctly to interpret and apply the manifold laws of the gentle science of arms. In this respect this book is largely a compilation, as are all modern works on the subject. It contains in addition a collection of material gathered from the use of royal and other seals upon colonial documents, and individual coat armor upon old tombstones, hatchments, tablets, family plate, wills, deeds, etc., showing an early practice and wide recognition of heraldry in America. It also presents a view of the present practical application of heraldry in the United States, particularly to the use of official, corporate, and personal seals, and insignia of orders and societies.

